# Characteristics and outcomes of acute-on-chronic liver failure patients with or without cirrhosis using two criteria

**DOI:** 10.1038/s41598-020-65529-5

**Published:** 2020-05-22

**Authors:** Xiaotian Dong, Jianqin He, Wenyi Chen, Rong Su, Yanping Xu, Xinyu Sheng, Lanjuan Li, Hongcui Cao

**Affiliations:** 1 Department of Laboratory Medicine, The First Affiliated Hospital, College of Medicine, Zhejiang University, 79 Qingchun Rd, Hangzhou City, 310003 China; 2 State Key Laboratory for Diagnosis and Treatment of Infectious Diseases, The First Affiliated Hospital, College of Medicine, Zhejiang University, 79 Qingchun Rd, Hangzhou City, 310003 China; 3 National Clinical Research Center for Infectious Diseases, 79 Qingchun Rd, Hangzhou City, 310003 China; 4 Zhejiang Provincial Key Laboratory for Diagnosis and Treatment of Aging and Physic-chemical Injury Diseases, 79 Qingchun Rd, Hangzhou City, 310003 China

**Keywords:** Gastroenterology, Medical research, Diseases, Infectious diseases

## Abstract

The aim of the study was to identify the characteristics and outcomes in acute-on-chronic liver failure (ACLF) patients with or without cirrhosis using two criteria. Patients with acute deterioration of chronic hepatic disease or acute decompensation of cirrhosis were included retrospectively from April 10, 2016 to April 10, 2019. European Association for the Study of the Liver-chronic liver failure (EASL-CLIF) criterion except for consideration of cirrhosis and Chinese Group on the Study of Severe Hepatitis B (COSSH) criterion were used. Clinical features, laboratory data and survival curves were compared between the ACLF patients with and without cirrhosis. A total of 799 patients were included. Among them, 328 had COSSH and EASL ACLF, 197 had COSSH alone, and 104 had EASL alone. There were 11.6% more ACLF with COSSH criterion. Furthermore, EASL ACLF patients with non-cirrhosis vs. cirrhosis had different laboratory characteristics: ALT (423 vs. 154, *p* < 0.001), AST (303 vs. 157, *p* < 0.001), γ-GT (86 vs. 75, *p* < 0.01), and INR (2.7 vs. 2.6, *p* < 0.001) were significantly higher but creatinine (71 vs. 77, *p* < 0.01) were significantly lower; but importantly there was no statistical changes between non-cirrhosis and cirrhosis in EASL ACLF patients on 28-day (*p* = 0.398) and 90-day (*p* = 0.376) survival curves. However, 90-day (*p* = 0.030) survival curve was different between non-cirrhosis and cirrhosis in COSSH ACLF patients. COSSH ACLF score (auROC = 0.778 or 0.792, 95%CI 0.706–0.839 or 0.721–0.851) displayed the better prognostic ability for EASL ACLF patients with non-cirrhosis, but CLIF-C ACLF score (auROC = 0.757 or 0.796, 95%CI 0.701–0.807 or 0.743–0.843) still was the best prognostic scoring system in EASL ACLF patients with cirrhosis. In conclusions, EASL definition exhibited better performance on homogeneous identification of ACLF regardless of cirrhosis or non-cirrhosis. And COSSH ACLF score displayed the better prognostic ability for EASL ACLF patients without cirrhosis.

## Introduction

Acute-on-chronic liver failure (ACLF) is a syndrome with high 28-day and 90-day mortality rates^1^ where patients with chronic hepatic disease or cirrhosis undergo acute liver deterioration. Over the last decades, various ACLF definitions have been proposed by East and West organizations. Specific definitions were provided by the Asian Pacific Association for the Study of the Liver (APASL)^2,3^ and the World Gastroenterology Organization (WGO)^4^ in corresponding to experts’ consensus while the North American Consortium for the Study of End-Stage Liver Disease (NACSELD) Consortium^5^ and the European Association for the Study of the Liver-chronic liver failure (EASL-CLIF) Consortium^6^ defined the term based on prospective and observational study. After that, the Chinese Group on the Study of Severe Hepatitis B (COSSH) proposed a new HBV-ACLF criterion^7^ based on prospective study of 13 liver centers in China. Unfortunately, no definition can encompass all ACLF patients from the East and West, except incomplete WGO definition^8^. In this study, the EASL-CLIF definition was used because of its superior abilities for defining ACLF and predicting outcome^9^. Moreover, the ACLF patients were also defined using the COSSH criterion, for comparability with EASL-CLIF definition.

The clinicopathological characteristics of ACLF patients with cirrhosis have been detailedly evaluated in cohorts from East and West^6,10–12^. However, despite the large population of non-cirrhotic ACLF patients in China^13^, the features and outcomes of these patients were hardly investigated. Thus, in this retrospective study, we identified clinical features of ACLF patients without cirrhosis and explored the difference between the ACLF patients with or without cirrhosis through two criteria.

## Patients and Methods

### Patients

Patients (Age >18 years) with acute decompensation (encephalopathy, ascites, upper gastrointestinal [GI] hemorrhage or bacterial infection) of cirrhosis or severe liver injury (total bilirubin [TB] ≥ 5 mg/dL and international normalized ratio [INR] ≥ 1.5) of non-cirrhotic chronic liver disease^7^ between April 10, 2016 and April 10, 2019 in the First Affiliated Hospital, Zhejiang University were screened. Cirrhosis was identified according to the results of liver biopsy, endoscopic signs of portal hypertension, previous decompensation evidence, radiological liver nodularity image and laboratory data^12^. Hepatic encephalopathy (HE) was graded according to the West Haven criteria^14^. Ascites was detected by ultrasonography^15^. Bacterial infection was diagnosed as previously described^12^. ACLF was diagnosed by EASL-CLIF definition based on CLIF-SOFA score^6^, and COSSH criteria^7^.

Patients were excluded when (1) hospitalized for only 1 day; (2) were pregnant; (3) had Acquired Immune Deficiency Syndrome; (4) had hepatocellular carcinoma; (5) had other tumors; (6) received a liver transplant; (7) had incomplete laboratory data. The study complied the Declaration of Helsinki. All experimental protocols were approved by the Ethics Committee on Clinical Research of the First Affiliated Hospital, Zhejiang University and were carried out in accordance with the approved guidelines. Informed written consent was waived due to its retrospective nature.

### Data gathering

The subsequent information was gathered: general clinical records (age, sex, blood pressure, etiology, cirrhosis or non-cirrhosis), complications (HE, ascites, upper GI hemorrhage or bacterial infection), laboratory parameters and survival data. The whole data were gathered when ACLF occurried on clinical presentation or in time of hospitalization. Survival data were collected according to the medical records and outpatient information.

### Study design

The clinical characteristics, laboratory data as well as mortality were contrasted using two criteria between (i) ACLF and non ACLF patients in all enrolled patients, (ii) cirrhotic ACLF patients and non-cirrhotic ACLF patients.

### Statistical analysis

Categorical variables were compared by chi-square test and expressed as frequencies and percentages. Continuous variables were compared by Student’s t test or Mann-Whitney U test and presented as median (IQR). Survival curves were assessed through Log-rank test. The area under the receiver operating curve (auROC) of different prognostic scoring systems, including COSSH ACLF score (COSSH ACLFs)^7^, CLIF Consortium ACLF score (CLIF-C ACLFs)^16^, CLIF-sequential organ failure assessment (CLIF-SOFA) score^6^, Model for End-Stage Liver Disease (MELD)^17^, MELD-sodium (MELD-Na)^18^, and the integrated MELD (iMELD)^19^, were computed and evaluated through Z test (Delong’s method). Statistical analyses were accomplished by SPSS (version 21.0; IBM Corp., Armonk, NY, USA), GraphPad Prism (version 7; GraphPad Software Inc., San Diego, CA), and MedCalc software (MedCalc Software, Belgium).

## Results

### Different groups of patients

**Figure 1:**
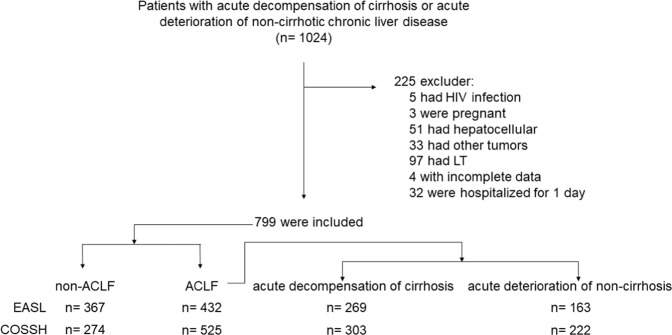
Distribution of patients with ACLF and non ACLF using EASL and COSSH criteria. *Abbreviation*: ACLF, acute-on-chronic liver failure; LT, liver transplantation; EASL, European Association for the Study of the Liver; COSSH, Chinese Group on the Study of Severe Hepatitis B.

A total of 799 patients who developed acute decompensation (AD) of cirrhosis and acute liver deterioration (ALD) of non-cirrhotic chronic hepatic disease were included after excluding 225 patients (Fig. 1). Among them, 328 developed COSSH- and EASL- defined ACLF, 197 developed COSSH-defined ACLF (COSSH ACLF) alone, and 104 developed EASL-defined ACLF (EASL ACLF) alone. The incidence rate for COSSH ACLF and EASL ACLF was 65.7% (525/799) and 54.1% (432/799), respectively. There were 11.6% more of ACLF cases when defined by COSSH criteria.

### EASL ACLF was more severe and with higher short time mortality than COSSH ACLF

**Figure 2:**
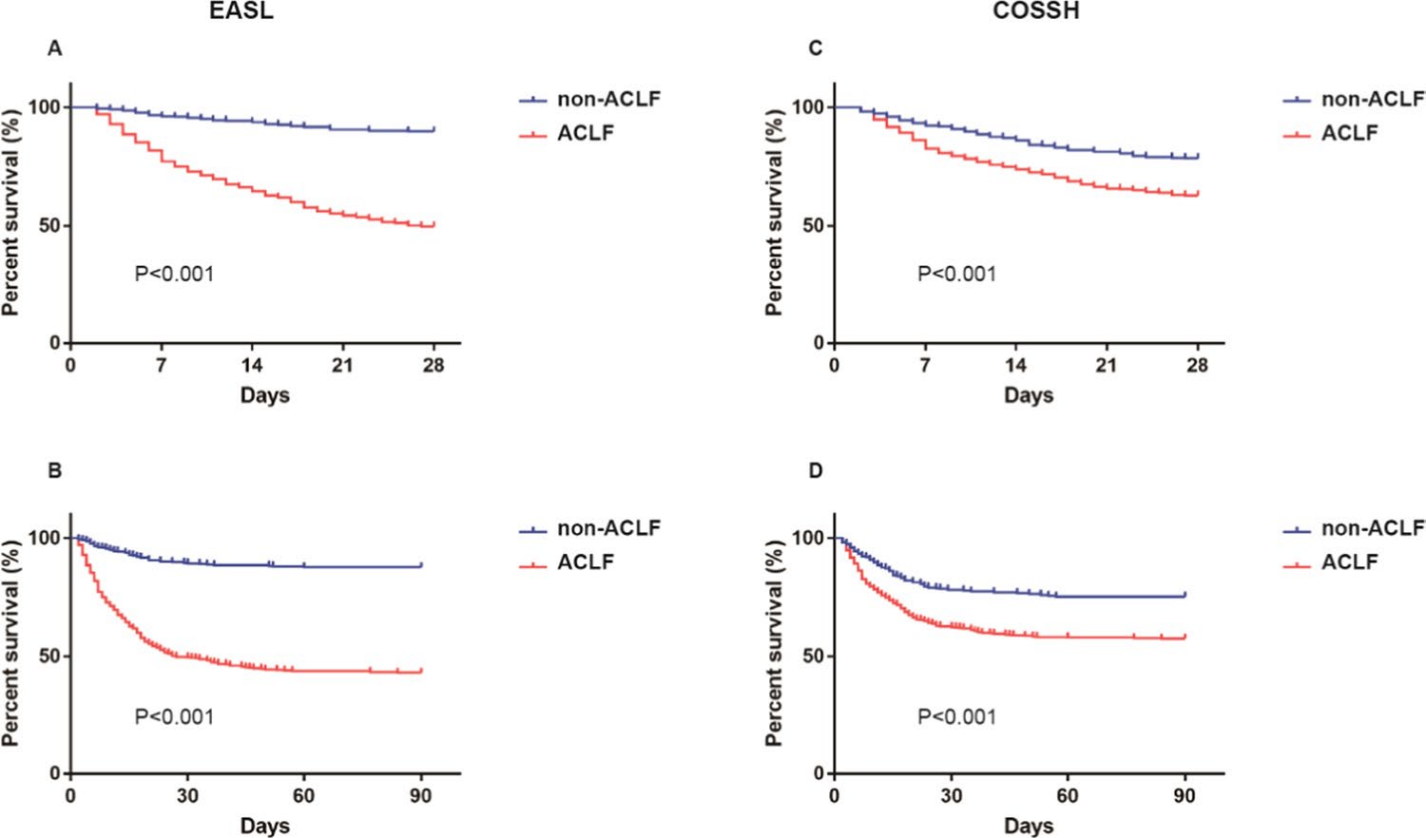
28-day and 90-day survival curves of ACLF and non-ACLF patients using EASL and COSSH criteria and Log-rank test were used to compare two groups. *Abbreviation*: ACLF, acute-on-chronic liver failure; EASL, European Association for the Study of the Liver; COSSH, Chinese Group on the Study of Severe Hepatitis B.

The detailed comparision of the characteristics between EASL ACLF and COSSH ACLF patients was displayed in Table 1. ACLF and non-ACLF patients were mainly HBV carriers, and ACLF patients were older than non-ACLF patients. In addition, ADs occurred more frequently in ACLF patients although there was no discrepancy between ACLF and non- ACLF patients in the prevalence of cirrhosis. Compared to non ACLF patients, the levels of TB, INR, WBC count and ferritin were significantly higher while γ-GT and serum sodium were significantly lower in EASL ACLF and COSSH ACLF patients. Liver and coagulation failure were most commonly seen in ACLF patients defined by two criteria. Six prognostic scoring systems indicated a worse outcome for EASL ACLF and COSSH ACLF patients than non-ACLF patients, in accordance with 28-day and 90-day survival curves (Table 1, Fig. 2).

**Table 1:** Characteristics of patients with ACLF and non-ACLF.

Characteristic	EASL		COSSH		*p* value
	Non-ACLF (n = 367)	ACLF (n = 432)	Non-ACLF (n = 274)	ACLF (n = 525)	
Age (years)	48.0 (19.0)	51.0 (19.0)*	49.5 (21.0)	50.0 (18.0)	0.092
Male, no. (%)	309 (84.2)	338 (78.2)*	214 (78.1)	433 (82.5)	0.099
**Aetiology**					
HBV, no. (%)	328 (89.4)	351 (81.2)*	229 (83.6)	450 (85.7)	0.063
Alcohol, no. (%)	17 (4.6)	26 (6.0)	22 (8.0)	21 (4.0)^†^	0.150
HBV + Alcohol, no. (%)	4 (1.1)	16 (3.7)*	2 (0.7)	18 (3.4)^†^	0.819
Others, no. (%)	18 (4.9)	39 (9.0)*	21 (7.7)	36 (6.9)	0.214
**Complications**					
Ascites, no. (%)	281 (76.6)	364 (84.3)*	209 (76.3)	436 (83.0)^†^	0.614
GI hemorrhage, no. (%)	18 (4.9)	60 (13.9)*	29 (10.6)	49 (9.3)	0.027^§^
Bacterial infection, no. (%)	47 (12.8)	84 (19.4)*	47 (17.2)	84 (16.0)	0.163
**Laboratory data**					
Albumin, g/L	31.3 (6.1)	30.8 (6.1)	30.8 (6.8)	31.1 (5.8)	0.237
ALT, U/L	191.0 (420.0)	230.0 (533.0)	175.0 (519.5)	229.0 (482.5)^†^	0.897
AST, U/L	144.0 (288.5)	190.0 (365.0)*	146.0 (374.8)	173.0 (302.5)	0.364
ALP, U/L	131.0 (46.0)	134.0 (56.0)	125.0 (53.5)	136.0 (50.5)^†^	0.334
TB, μmol/L	258.0 (200.8)	372.0 (219.2)*	161.8 (80.5)	358.0 (157.8)^†^	0.177
γ-GT, U/L	94.0 (83.0)	78.0 (74.0)*	101.5 (88.5)	78.0 (74.0)^†^	0.945
Creatinine, μmol/L	65.0 (19.0)	75.0 (49.0)*	66.5 (24.0)	67.0 (23.0)	<0.001^§^
Sodium, mmol/L	138.0 (4.0)	137.0 (6.0)*	138.0 (5.3)	137.0 (4.0)^†^	0.858
INR	1.8 (0.4)	2.6 (1.0)*	1.8 (0.7)	2.1 (0.8)^†^	<0.001^§^
WBC, 10^9^/L	6.0 (3.4)	7.0 (4.6)*	6.0 (3.5)	6.8 (4.2)^†^	0.282
Hemoglobin, g/L	126.0 (26.5)	121.0 (31.0)	121.5 (33.0)	125.0 (27.0)^†^	0.071
Hematocrit, %	35.8 (8.3)	34.8 (9.0)*	35.1 (10.1)	35.4 (8.5)	0.221
Platelet, 10^9^/L	101.0 (72.5)	99.0 (74.0)	100.5 (81.3)	100.0 (70.0)	0.622
C reactive protein, mg/L	11.9 (10.5)	12.0 (12.5)	11.9 (15.4)	12.0 (10.5)	0.921
Alpha fetoprotein, μg/L	89.7 (256.5)	42.4 (131.8)*	38.1 (224.1)	76.5 (211.4)^†^	<0.001^§^
Ferritin, μg/L	1779.1 (2411.7)	2653.9 (3799.8)*	1813.3 (2745.0)	2574.9 (3473.9)^†^	0.985
**Organ failure**					
Liver, no. (%)	268 (73.0)	411 (95.1)*	154 (56.2)	525 (100.0)^†^	<0.001^§^
Kidney, no. (%)	0 (0.0)	90 (20.8)*	19 (6.9)	71 (13.5)^†^	0.003^§^
Cerebral, no. (%)	2 (0.5)	100 (23.1)*	22 (8.0)	80 (15.2)^†^	0.002^§^
Coagulation, no. (%)	11 (3.0)	315 (72.9)*	78 (28.5)	248 (47.2)^†^	<0.001^§^
Circulation, no. (%)	1 (0.3)	72 (16.7)*	19 (6.9)	54 (10.3)	0.004^§^
Lung, no. (%)	0 (0.0)	62 (14.4)*	15 (5.5)	47 (8.9)	0.009^§^
Hepatic encephalopathy grade I or II	12 (3.3)	143 (33.1)*	31 (11.3)	59 (11.2)	<0.001§
**Severity score**					
COSSH ACLFs	5.2 (0.6)	6.3 (1.5)*	5.4 (1.0)	5.8 (1.3)^†^	<0.001^§^
CLIF-C ACLFs	37.7 (8.7)	49.6 (15.0)*	37.5 (12.1)	44.2 (13.9)^†^	<0.001^§^
CLIF-SOFA	8.0 (1.0)	11.0 (3.0)*	8.0 (2.0)	10.0 (2.0)^†^	<0.001^§^
MELD	19.9 (5.4)	27.4 (7.7)*	18.1 (6.1)	23.5 (6.4)^†^	0.109
MELD-Na	21.4 (5.4)	28.4 (7.7)*	19.6 (5.7)	25.2 (6.0)^†^	0.108
iMELD	3.5 (0.9)	5.8 (4.2)*	3.6 (1.8)	4.3 (2.8)	<0.001^§^
**Transplant-free mortality**					
28-day, no. (%)	37 (10.1)	217 (50.2)*	59 (21.5)	195 (37.1)^†^	<0.001^§^
90-day, no. (%)	44 (12.0)	247 (57.2)*	68 (24.8)	223 (42.5)^†^	<0.001^§^
Cirrhosis	210 (57.2)	269 (62.3)	176 (64.2)	303 (57.7)	0.153

^§^p < 0.05, ACLF patients, EASL-ACLF vs. COSSH-ACLF.

*p < 0.05, patients with EASL definition, Non-ACLF vs. ACLF.

^†^p < 0.05, patients with COSSH definition, Non-ACLF vs. ACLF.

Moreover, compared to COSSH ACLF patients, the levels of Creatinine and INR were significantly higher while alpha fetoprotein was significantly lower in EASL ACLF. Organ failures, except for liver failure, occured more frequently in EASL ACLF compared to COSSH ACLF. Four prognostic scoring systems indicated a worse outcome for EASL ACLF than COSSH ACLF patients, consistent with 28-day and 90-day survival rates (Table 1).

### EASL ACLF patients with cirrhosis and non-cirrhosis had a more consistent outcome

**Figure 3:**
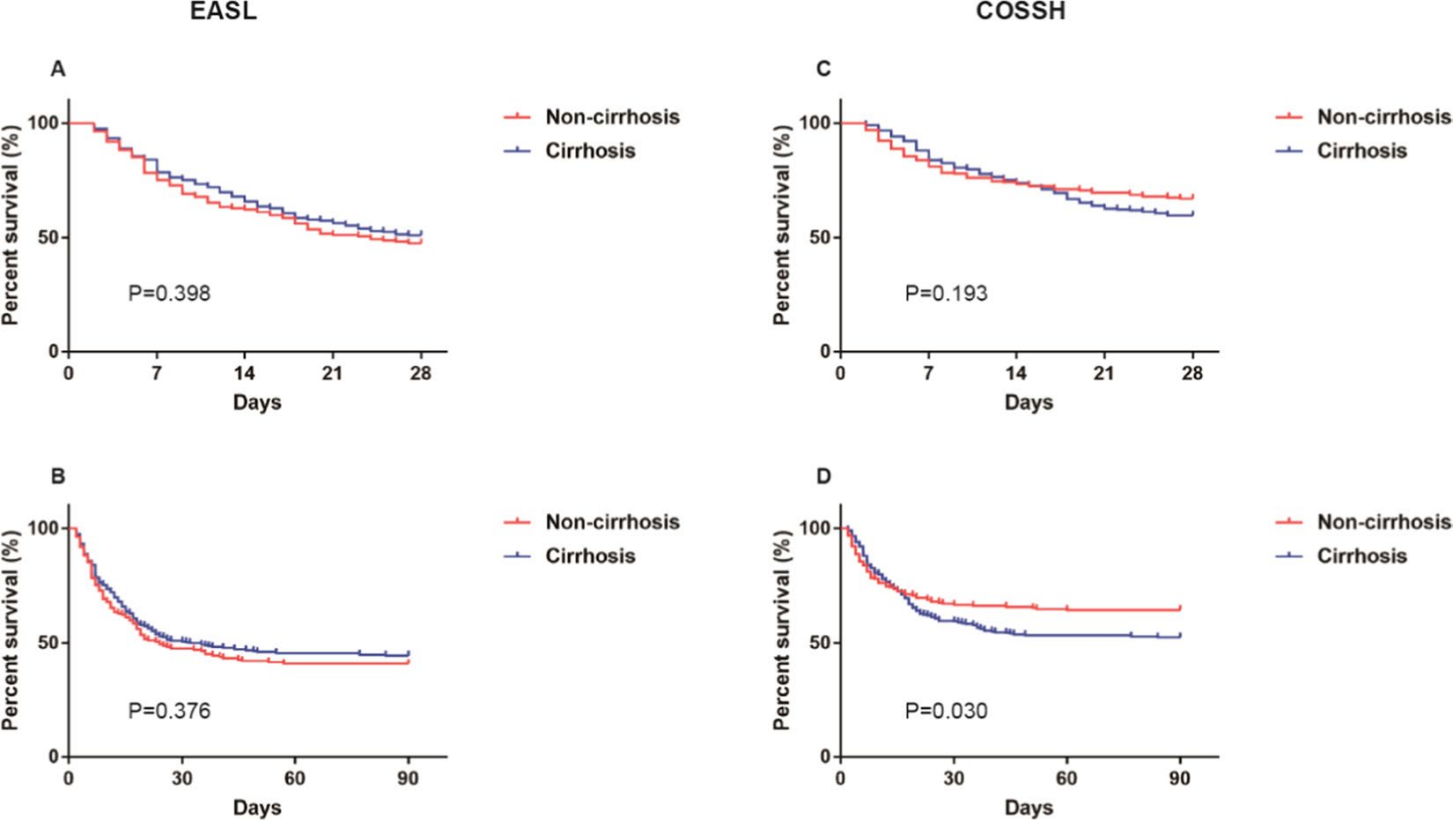
28-day and 90-day survival curves of ACLF patients with cirrhosis and non-cirrhosis using EASL and COSSH criteria and Log-rank test were used to compare two groups. *Abbreviation*: ACLF, acute-on-chronic liver failure; EASL, European Association for the Study of the Liver; COSSH, Chinese Group on the Study of Severe Hepatitis B.

The detail of characteristics between ACLF patients with cirrhosis and non-cirrhosis was compared in Table 2. EASL ACLF and COSSH ACLF patients with non-cirrhosis were younger and had more HBV infection than cirrhotic EASL ACLF and COSSH ACLF patients. But ADs were happened more commonly in both EASL ACLF and COSSH ACLF patients with cirrhosis. The measures of albumin, ALT, AST, γ-GT, serum sodium, WBC count, hemoglobin, hematocrit, platelet count, alpha fetoprotein and ferritin were significantly higher but c reactive protein were significantly lower in EASL ACLF and COSSH ACLF patients with non-cirrhosis, compared with ACLF patients with cirrhosis. In addition, lower occurrence of kidney failure was observed in EASL ACLF and COSSH ACLF patients with non-cirrhosis, compared with ACLF patients with cirrhosis. Six prognostic scoring systems predicted no statistical difference in outcomes between EASL ACLF patient with cirrhosis and non-cirrhosis. And there was also no statistical alteration between EASL ACLF patient with cirrhosis and non-cirrhosis on 28-day and 90-day survival curves (Fig. 3). However, COSSH ACLF score, CLIF-C ACLF score and iMELD score indicated a worse outcome for COSSH ACLF patients with cirrhosis than non-cirrhosis, and 90-day survival curves were consistent with that (Table 2, Fig. 3).

**Table 2:** Characteristics of ACLF patients with cirrhosis and non-cirrhosis.

Characteristic	EASL ACLF		COSSH ACLF		*p* value
	Cirrhosis (n = 269)	Non-cirrhosis (n = 163)	Cirrhosis (n = 303)	Non-cirrhosis (n = 222)	
Age (years)	53 (17)	46 (18)*	53 (16)	45 (19)^†^	0.498
Male, no. (%)	204 (75.8)	134 (82.2)	238 (78.5)	195 (87.8)^†^	0.122
**Aetiology**					
HBV, no. (%)	197 (73.2)	154 (94.5)*	234 (77.2)	216 (97.3)^†^	0.158
Alcohol, no. (%)	24 (8.9)	2 (1.2)*	20 (6.6)	1 (0.5)^†^	0.576
HBV + Alcohol, no. (%)	14 (5.2)	2 (1.2)*	16 (5.3)	2 (0.9)^†^	1.000
Others, no. (%)	34 (12.6)	5 (3.1)*	33 (10.9)	3 (1.4)^†^	0.291
**Complications**					
Ascites, no. (%)	255 (94.8)	109 (66.9)*	289 (95.4)	147 (66.2)^†^	0.893
GI hemorrhage, no. (%)	49 (18.2)	11 (6.7)*	39 (12.9)	10 (4.5)^†^	0.338
Bacterial infection, no. (%)	67 (24.9)	17 (10.4)*	67 (22.1)	17 (7.7)^†^	0.344
**Laboratory data**					
Albumin, g/L	30.2 (5.5)	31.9 (5.7)*	30.9 (5.7)	31.7 (5.6)^†^	0.678
ALT, U/L	154.0 (356.0)	423.0 (692.0)*	149.0 (328.0)	401.5 (608.3)^†^	0.365
AST, U/L	157.0 (253.0)	303.0 (404.0)*	148.0 (213.0)	234.5 (362.3)^†^	0.193
ALP, U/L	131.5 (57.0)	140.0 (55.0)	135.0 (50.0)	137.0 (50.3)	0.829
TB, μmol/L	371.5 (239.2)	375.0 (189.0)	368.0 (167.0)	342.1 (145.5)^†^	0.025^§^
γ-GT, U/L	75.0 (67.8)	86.0 (74.0)*	74.0 (57.0)	87.5 (78.5)^†^	0.772
Creatinine, μmol/L	77.0 (60.5)	71.0 (43.0)*	67.0 (25.0)^#^	64.0 (19.3)	<0.001^§^
Sodium, mmol/L	137.0 (6.0)	138.0 (4.0)*	137.0 (5.0)	138.0 (4.0)^†^	0.693
INR	2.6 (1.0)	2.7 (1.0)*	2.1 (0.7)^#^	2.1 (0.9)	<0.001^§^
WBC, 10^9^/L	6.8 (4.2)	7.4 (4.8)*	6.5 (4.1)	7.1 (3.8)^†^	0.167
Hemoglobin, g/L	116.0 (29.0)	132.0 (28.0)*	119.0 (26.0)	133.0 (22.3)^†^	0.346
Hematocrit, %	33.5 (8.1)	38.0 (8.5)*	34.1 (7.1)	38.0 (7.1)^†^	0.837
Platelet, 10^9^/L	84.0 (69.0)	119.0 (73.0)*	86.0 (68.0)	118.5 (70.5)^†^	0.694
C reactive protein, mg/L	13.8 (13.9)	10.2 (8.1)*	13.2 (11.2)	10.7 (8.8)^†^	0.290
Alpha fetoprotein, μg/L	37.7 (112.8)	53.9 (189.3)*	59.9 (151.1)^#^	109.3 (235.5)^†^	0.001^§^
Ferritin, μg/L	2098.3 (3288.8)	3404.0 (3934.0)*	2115.7 (3036.2)	3105.4 (3918.9)^†^	0.328
**Organ faliure**					
Liver, no. (%)	253 (94.1)	158 (96.9)	303 (100.0)^#^	222 (100.0)	0.013^§^
Kidney, no. (%)	66 (24.5)	24 (14.7)*	50 (16.5)^#^	21 (9.5)^†^	0.112
Cerebral, no. (%)	57 (21.2)	43 (26.4)	44 (14.5)^#^	36 (16.2)	0.015^§^
Coagulation, no. (%)	186 (69.1)	129 (79.1)*	145 (47.9)^#^	103 (46.4)	<0.001^§^
Circulation, no. (%)	47 (17.5)	25 (15.3)	33 (10.9)^#^	21 (9.5)	0.079
Lung, no. (%)	36 (13.4)	26 (16.0)	25 (8.3)^#^	22 (9.9)	0.036^§^
Hepatic encephalopathy grade I or II	94 (34.9)	49 (30.1)	67 (22.1)^#^	61 (27.5)	0.579
**Severity score**					
COSSH ACLFs	6.3 (1.4)	6.2 (1.7)	5.9 (1.2)^#^	5.7 (1.4)^†^	<0.001^§^
CLIF-C ACLFs	50.9 (14.4)	48 (16.7)	45.4 (13.5)^#^	42.3 (13.2)^†^	0.002^§^
CLIF-SOFA	11.0 (3.0)	11.0 (3.0)	10.0 (2.0)^#^	9.0 (2.0)	<0.001^§^
MELD	27.3 (8.4)	27.7 (6.9)	23.5 (6.6)	23.5 (5.9)	0.019^§^
MELD-Na	28.3 (7.9)	28.6 (6.9)	25.3 (6.1)	25.0 (6.2)	0.022^§^
iMELD	5.8 (4.1)	5.9 (4.7)	4.4 (2.5)^#^	4.2 (3.4)^†^	<0.001^§^
**Transplant-free mortality**					
28-day, no. (%)	139 (51.7)	78 (47.9)	122 (40.3)^#^	73 (32.9)	0.003^§^
90-day, no. (%)	166 (61.7)	81 (49.7)*	144 (47.5)^#^	79 (35.6)	0.006^§^

^§^p < 0.05, ACLF patients with Non-cirrhosis, EASL ACLF with Non-cirrhosis vs. COSSH ACLF with Non-cirrhosis.

^#^p < 0.05, ACLF patients with Cirrhosis, EASL ACLF with Cirrhosis vs. COSSH ACLF with Cirrhosis.

*p < 0.05, ACLF patients with EASL definition, Cirrhosis vs. Non-cirrhosis.

^†^p < 0.05, ACLF patients with COSSH definition, Cirrhosis vs. Non-cirrhosis.

### CLIF-C ALCF score was better in predicting ACLF patients with cirrhosis short time mortality, but COSSH ACLF score was better for ACLF patients with non-cirrhosis

In all EASL ACLF patients and EASL ACLF patients with non-cirrhosis, COSSH ACLFs possessed the best predictive value of 28-day and 90-day mortality among six prognostic scoring systems (Table 3). And CLIF-C ACLFs, CLIF-SOFA and iMELD scores also had good predictive value in those patients. However, CLIF-C ALCFs still was the best prognostic scoring system in EASL ACLF patients with cirrhosis. Furthermore, COSSH ACLFs, CLIF-SOFA and iMELD also had a well performance in prediction of these patients’ outcomes.

**Table 3:** Predictive value of six prognostic scoring systems in ACLF patients.

	28-day				90-day			
	auROC	95% CI	Z value	*p* value	auROC	95% CI	Z value	*p* value
**All ACLF patients**								
COSSH ACLFs	0.778	0.706–0.839			0.792	0.721–0.851		
CLIF-C ACLFs	0.754	0.680–0.818	0.891	0.373	0.765	0.692–0.828	0.983	0.326
CLIF-SOFA	0.765	0.692–0.828	0.479	0.632	0.778	0.706–0.839	0.518	0.604
MELD	0.605	0.525–0.680	3.676	<0.001	0.602	0.523–0.678	4.088	<0.001
MELD-Na	0.620	0.541–0.695	3.474	<0.001	0.616	0.537–0.691	3.926	<0.001
iMELD	0.761	0.688–0.824	0.521	0.602	0.766	0.693–0.828	0.803	0.422
**ACLF patients with cirrhosis**								
COSSH ACLFs	0.726	0.669–0.779			0.767	0.712–0.816		
CLIF-C ACLFs	0.757	0.701–0.807	1.304	0.192	0.796	0.743–0.843	1.213	0.225
CLIF-SOFA	0.740	0.683–0.791	0.550	0.582	0.787	0.733–0.834	0.782	0.434
MELD	0.612	0.551–0.671	2.878	0.004	0.581	0.520–0.641	4.659	<0.001
MELD-Na	0.624	0.563–0.682	2.545	0.011	0.590	0.528–0.649	4.357	<0.001
iMELD	0.753	0.697–0.803	0.881	0.378	0.748	0.692–0.799	0.619	0.536
**ACLF patients without cirrhosis**								
COSSH ACLFs	0.778	0.706–0.839			0.792	0.721–0.851		
CLIF-C ACLFs	0.754	0.680–0.818	0.891	0.373	0.765	0.692–0.828	0.983	0.326
CLIF-SOFA	0.765	0.692–0.828	0.479	0.632	0.778	0.706–0.839	0.518	0.604
MELD	0.605	0.525–0.680	3.676	<0.001	0.602	0.523–0.678	4.088	<0.001
MELD-Na	0.620	0.541–0.695	3.474	<0.001	0.616	0.537–0.691	3.926	<0.001
iMELD	0.761	0.688–0.824	0.521	0.602	0.766	0.693–0.828	0.803	0.422

Data were compared by Z test (Delong’s method)

## Discussion

ACLF is a syndrome accompanied by multisystem organ failure and high 28-day and 90-day mortality. The cause of ACLF is dissimilar in the East and West. The East ACLF patients are primarily developed from the viral (hepatitis B or C) related chronic hepatic disease (with or without cirrhosis)^20^. And various HBV-ACLF related prognostic models based on serum miRNAs or multicenter data were established^7,21^. In this study, we attempted to obtain the variance between the ACLF patients with and without cirrhosis using the two definitions (EASL-CLIF definition and COSSH definition), and also verified which one was more appropriate definition for ACLF patients.

Our study indicated that ACLF had similar prevalence in patients with cirrhosis and non-cirrhosis using two definitions (Fig. 1). And, coagulation failure was the most common organ failure in our ACLF patients (EASL ACLF and COSSH ACLF), except for liver failure, which was different with the CANONIC study^6^. In addition, ACLF patients were older and had more severe deterioration of laboratory parameters than non-ACLF patients, which paralleled the outcomes of ACLF patients. However, EASL ACLF patients had more severe kidney function and coagulation function (higher level of creatinine and INR) accompanied by higher prognostic scores and worse outcomes, compared with COSSH ACLF patients. These results indicated that COSSH definition improved the sensitivity for finding more ACLF patients (11.6%) but also reduced some important characteristics of ACLF patients, for example supposedly worse kidney and coagulation function.

Importantly, although EASL ACLF and COSSH ACLF patients with non-cirrhosis both had distinct characteristics with ACLF patients with cirrhosis, but similar outcomes and prognostic scores of ACLF patients with cirrhosis and non-cirrhosis were observed only in EASL definition (Table 2, Fig. 3). These data indicated that COSSH ACLF patients with non-cirrhosis exhibited higher levels of ALT and AST but relatively lower level of TB, compared with COSSH ACLF patients with cirrhosis. In addition, COSSH ACLF patients with non-cirrhosis exhibited similar level of creatinine with COSSH ACLF cirrhosis patients, but higher proportion of kidney failure was observed in COSSH ACLF cirrhosis patients. However, EASL ACLF patients with non-cirrhosis exhibited worse liver function (higher levels of ALT and AST) and coagulation function (higher level of INR) but relatively better kidney function (lower level of creatinine) than EASL ACLF patients with cirrhosis. In addition, EASL ACLF patients with non-cirrhosis were younger and exhibited higher occurrence of coagulation failure and lower occurrence of kidney failure and ADs. These results indicated our EASL ACLF patients with non-cirrhosis might also meet APASL definition (TB ≥ 5 mg/dL and INR ≥ 1.5 complicated within 4 weeks by clinical ascites and/or encephalopathy)^22^. Actually, in 163 EASL ACLF patients with non-cirrhosis, 143 (87.7%) developed APASL and EASL ACLF in our study. This result verified that EASL definition also had good performance on diagnosis of ACLF patients with non-cirrhosis. Importantly, EASL ACLF patients with cirrhosis and non-cirrhosis had a more consistent prognostic score and outcome. Moreover, both EASL ACLF patients with and without cirrhosis were possessed similar relatively high occurrence of liver failure. Thus, the development of ACLF patients was highly determined by the liver function and EASL definition exhibited better performance on homogeneous identification of ACLF.

ACLF patients always exhibit one or more organ failures and have high mortality rates. In our study, the short time mortality of EASL ACLF patients with and without cirrhosis are similar to other studies^7,12^. And there was no statistical difference between EASL ACLF patients with and without cirrhosis on 28-day and 90-day survival curves (Fig. 3). Furthermore, COSSH ACLF score (0.741 × INR + 0.523 × HBV-SOFA + 0.026 × age + 0.003 × TB)^7^, not CLIF-C ACLF score, had the best predictive value on the 28-day and 90-day mortality in ACLF patients with non-cirrhosis. Interestingly, iMELD score, as TB, creatinine, INR, age and HE are main element in iMELD score^19^, CLIF-SOFA and CLIF-C ACLF score also had well performance on predicting short time prognosis of ACLF patients with non-cirrhosis. However, CLIF-C ACLF score (10× [0.33 × CLIF-OFs + 0.04 × age + 0.63 ×ln (WBC count)-2) still was the best prognostic scoring system in EASL ACLF patients with cirrhosis, probably because age and systemic inflammation (high WBC count) were strongly associated with the worsen of ACLF patients with cirrhosis^16,23^.

Considering this is a single center study that potential patient selection bias may exist, multicenter prospective study was needed in the future. In summary, we identified EASL definition was better and observed the distinct characteristics but similar outcomes between EASL ACLF patients with and without cirrhosis. Moreover, COSSH ACLF score displayed the better prognostic ability for ACLF patients with non-cirrhosis, but CLIF-C ACLF score still was the best prognostic scoring system in EASL ACLF patients with cirrhosis.
